# Infantile Hemangiomas Masquerading as Other Periocular Disorders

**DOI:** 10.1155/2012/290645

**Published:** 2012-04-01

**Authors:** Jennifer Hsu, Brian G. Mohney

**Affiliations:** ^1^Mayo Medical School, Rochester, MN 55905, USA; ^2^Department of Ophthalmology, Mayo Clinic and Mayo Foundation, Rochester, MN 55905, USA

## Abstract

Periocular tumors are common in infancy. The most common periocular tumors are capillary hemangiomas, which are present in 1-2% of newborns and develop in 10%–12% of children by the age of 1 year old. Deep capillary hemangiomas may be more challenging to diagnose than superficial capillary hemangiomas and can be confused with other orbital lesions. Deep orbital hemangiomas can mimic teratoma, lymphangioma, rhabdomyosarcoma, metastatic neuroblastoma, and granulocytic sarcoma. In this paper, we describe 2 pediatric cases where previously diagnosed dermoid cyst and dacrocystocele were found to be capillary hemangiomas upon biopsy. Approaches to distinguish capillary hemangiomas from other periocular tumors are further discussed. To our knowledge, this is the first case report of periocular hemangiomas imitating a dermoid cyst and a dacrocystocele. These cases emphasize the importance of including infantile hemangiomas in the differential diagnosis of subcutaneous periocular abnormalities.

## 1. Introduction

Periocular tumors are common in infancy, the most prevalent being infantile hemangiomas, which occur on any part of the body in 1-2% of newborns [1]. While superficial hemangiomas are easily recognizable, deep lesions may be more challenging to diagnose and can, therefore, be confused with subcutaneous lesions. It is important to be aware that hemangiomas can mimic other periocular lesions and to keep the diagnosis of capillary hemangioma in the differential diagnosis of deep orbital lesions. In this paper, we describe 2 infants who were initially diagnosed as having other periocular lesions (dermoid cyst and dacrocystocele) before being diagnosed with infantile hemangiomas. Approaches to help recognize capillary hemangiomas from other periocular tumors are further discussed.

## 2. Report of Cases

### 2.1. Case 1

**Figure 1:**
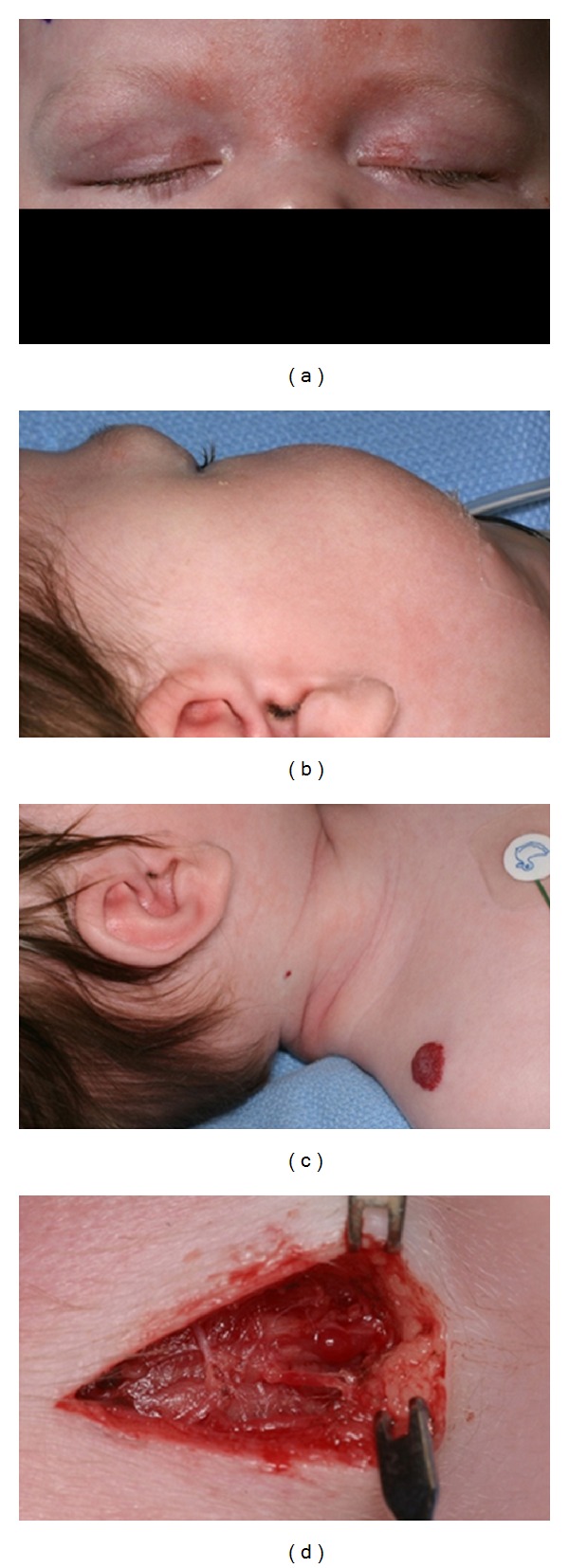
(a) Firm, oblong 1.5 × 1.0 cm sized rubbery mass over the right temporal brow. (b) Lesion found under the right brow. (c) Capillary hemangioma on the right shoulder and right neck. (d) Incision through the eyebrow on the temporal edge made to evaluate the tumor.

 An otherwise healthy three-month-old female presented to our department for a six-week history of a right temporal brow mass. Her vision and ocular examination were unremarkable except for a 1.5 × 1.0 cm firm, rubbery mass along the right temporal brow (Figures 1(a) and 1(b)). The palpated lesion appeared to be fixed to the underlying bone, and there was no palpable orbital rim defect. A diagnosis of a dermoid cyst was made, and the patient was scheduled for surgical excision.

In the surgical suite, the patient was noted to have a capillary hemangioma on the right shoulder and a smaller one on the right neck (Figure 1(c)). Because the palpated brow lesion was not classic for a dermoid cyst, a diagnosis of capillary hemangioma was considered. A 10 mm incision through the lateral eyebrow was made to evaluate the tumor (Figure 1(d)). An irregular reddish mass was observed, and, when palpated, the findings were consistent with an infantile hemangioma. One cc of 40 mg Kenalog was injected into the growth. The patient was seen in followup at three and six months postoperatively, at which time the lesion was essentially unchanged in size, associated with mild astigmatism (+4.50 + 1.50 × 115 OD; +5.00 + 1.50 × 90 OS) and no apparent amblyopia.

### 2.2. Case 2

**Figure 2:**
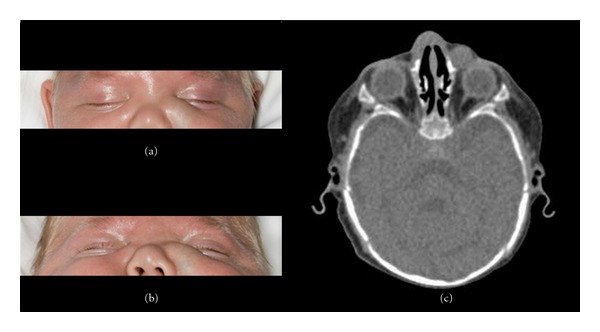
(a) and (b) Nonerythematous, bluish lesion 1.5 × 1.0 cm below the nasal lower lid. (c) CT scan showing 11 × 9 mm lesion along left nasocanthal fold.

A four-month-old male born at 27 weeks gestation was examined in the neonatal intensive care unit for a several-day history of a nontender, bluish subcutaneous 1.5 × 1.0 cm mass located below the left medial canthus (Figures 2(a) and 2(b)). There was mucopurulent discharge from the lower punctum upon palpation. He was diagnosed with a dacryocystocele, although other paramedian nasal lesions including a dermoid, encephalocele, or glioma were not excluded from the differential. A high-resolution CT scan of the paranasal sinuses and orbits was obtained (Figure 2(c)) and showed an 11 × 9 mm low-density lesion centered along the left nasocanthal fold extending from the left medial canthus to the left nasolabial fold. One month earlier, the mass had measured 4 × 4 mm by magnetic resonance imaging. Given the enlargement and location, the lesion most likely represented an enlarging dacryocystocele. 

A nasal canthus duct exploration was undertaken with otolaryngology assistance. The right nasal cavity was patent and had no evidence of cyst or purulent secretion, while the left nasal cavity had some mucoid crusting. No purulent debris was expressed with palpation of the mass, and there was no cystic component noted intranasally. The mass on the cheek and lateral nose was determined to have no communication with the nasal canthus passage and was thought to be more consistent with an infantile hemangioma. The child was started on systemic propanolol, after which a decrease in size was observed.

## 3. Comment

Capillary hemangiomas occur in approximately 1-2% of neonates and up to 10–12% of infants within the first year of life [1]. These common vascular tumors, which can occur both cutaneously and subcutaneously on any part of the body, can imitate other less common disorders. Orbital hemangiomas have been known to mimic a teratoma, lymphangioma, rhabdomyosarcoma, metastatic neuroblastoma, and granulocytic sarcoma [2]. Subcutaneous hemangiomas, however, are less commonly known to mimic other periocular disorders. These two cases, to our knowledge, are the first reports of capillary hemangiomas imitating a dacryocystocele and dermoid cyst. 

Capillary hemangiomas are sometimes seen at birth but appear more commonly within the first few weeks of life and may enlarge quickly over the first year, after which the tumor begins to involute. Depending on the depth and location of the tumor, the clinical appearance may vary. Superficial hemangiomas produce an elevated strawberry-colored nodule, while deep orbital hemangiomas typically present as a fluctuant, compressible bluish mass [2]. Infantile hemangiomas are likely to develop in the superonasal quadrant of the orbit and on the upper eyelid [3].

Dermoid cysts manifest as a round, firm, smooth, and nontender mass that may be mobile or fixed to the underlying periosteum. The mass will typically be a slowly enlarging and painless subcutaneous cyst located superonasally or superotemporally to the eye [4]. Dermoid cysts are usually congenital but may not be apparent at birth, becoming more evident during the first decade of life. Deeper orbital dermoid cysts will sometimes remain asymptomatic until later in life [4]. Congenital dacryocystoceles typically present in the first week or month of life and result from the blockage of the two valves at the opposing ends of the nasal canthus system, the valve of Hasner and the valve of Rosenmuller. Dacryocystocele present as a pink or blue subcutaneous swelling in the region below the medial canthus due to the accumulation of fluid in the lacrimal sac.

The diagnosis of periocular disorders is generally made on clinical examination alone. A careful history that includes age of onset, color, and location, along with physical examination findings is generally sufficient in determining the pathological condition [2]. In cases in which clinical examination alone is inconclusive, other diagnostic modalities such as ultrasonography, computed tomography (CT), and magnetic resonance imaging (MRI) may be utilized. On ultrasound, a hemangioma will appear as an irregularly shaped mass with heterogenous internal echoes while a dacroycystocele will appear as a cystic mass connected to a dilated nasal canthus duct with fluid and debris. MRI is advantageous in characterizing the contents of cysts without radiation exposure, while CT is advantageous in detecting changes in bone structure [5].

 In this paper, two distinct patients with periocular lesions, initially diagnosed as a dermoid cyst and dacryocystocele, were later found to have infantile hemangiomas. The prevalent occurrence and widespread distribution of infantile hemangiomas can simulate other periocular disorders. It is important to obtain a complete history and appropriate imaging to help make an accurate diagnosis. Infantile hemangiomas should be considered in the differential diagnosis of any subcutaneous periocular abnormalities.
